# Different Temporal Structure for Form versus Surface Cortical Color Systems – Evidence from Chromatic Non-Linear VEP

**DOI:** 10.1371/journal.pone.0015266

**Published:** 2010-12-20

**Authors:** David P. Crewther, Sheila G. Crewther

**Affiliations:** 1 Brain Sciences Institute, Swinburne University of Technology, Melbourne, Victoria, Australia; 2 School of Psychological Science, La Trobe University, Melbourne, Victoria, Australia; Chiba University Center for Forensic Mental Health, Japan

## Abstract

Physiological studies of color processing have typically measured responses to spatially varying chromatic stimuli such as gratings, while psychophysical studies of color include color naming, color and light, as well as spatial and temporal chromatic sensitivities. This raises the question of whether we have one or several cortical color processing systems. Here we show from non-linear analysis of human visual evoked potentials (VEP) the presence of distinct and independent temporal signatures for form and surface color processing. Surface color stimuli produced most power in the second order Wiener kernel, indicative of a slowly recovering neural system, while chromatic form stimulation produced most power in the first order kernel (showing rapid recovery). We find end-spectral saturation-dependent signals, easily separable from achromatic signals for surface color stimuli. However physiological responses to form color stimuli, though varying somewhat with saturation, showed similar waveform components. Lastly, the spectral dependence of surface and form color VEP was different, with the surface color responses almost vanishing with yellow-grey isoluminant stimulation whereas the form color VEP shows robust recordable signals across all hues. Thus, surface and form colored stimuli engage different neural systems within cortex, pointing to the need to establish their relative contributions under the diverse chromatic stimulus conditions used in the literature.

## Introduction

When we say “I want a red apple” it is clear that we are talking about the surface properties of the object. However, it is unclear when we describe the results of experiments involving red/green gratings whether we are talking about the contours that define the form or the colors that lie between, their borders of course, defining the edges. While it might seem obvious that the latter is the case, consideration of how knowledge of chromatic processing in the primate visual system has been derived might cause us to rethink.

The segregation of color from luminance processing starts in the retina where outputs of three different cone types plus rod photoreceptors combine to produce chromatically sensitive color-opponent cells (along red-green and blue-yellow dimensions, and color insensitive broad-band cells [1] reviewed [2]. However, the segregation of chromatic and achromatic processing is certainly not exclusive, with the color sensitive parvocellular ganglion cells well able to respond to luminance contrast.

Cortically, the red–green color-opponent signal of the primate parvocellular system is relayed to layers 4Cβ and 6 of Area V1 [3], [4], while blue-yellow opponent signals project to cytochrome oxidase rich blobs of layer 2/3 in V1 [3], [5] via the koniocellular system. The blob-like patches of color response align well with anatomically defined cytochrome oxidase rich blobs, and while located centrally on ocular dominance columns, do not overlap with centres of orientation-selective domains [6].

Single cell recordings from monkey cortex demonstrate that most cortical neurons prefer modulation along directions in color space lying close to the achromatic axis and tend to be orientation selective, while the color sensitive cortical cells (at least in areas V1 and V2) tend to be more poorly oriented and produce the same response to spatially uniform color stimulation as to gratings [7], [8]. One role for V1 and V2 appears to be the extraction of form information for the recognition of objects further along the visual pathway and to this end early cortical neurons have been implicated in border ownership of objects [9]. Conway et al [10] found populations of neurons in monkey that either responded more strongly to temporal alternation of color (possibly coding for hue) or spatial adjacency of color (likely feeding form recognition circuits), providing the basis for the influence of form on color perception [11].

Early recordings of human visual evoked potential (VEP) responses [12] concluded that ‘spatial contrast, not color, is the only relevant attribute to the stimuli’. Different color specific components in the VEP have been identified using various stimulation modes. The VEPs evoked by black/red or black/green grating stimulation are similar, presumably reflecting achromatic channel activity, while isoluminant stimulation produces a color-dependent signal [13] with the red/green negativity recorded in normals, vanishing in color defective subjects around isoluminance [14], though see [15]. The use of unstructured equibright color stimuli allowed Paulus and co-workers to avoid complications of form stimulation, finding the presence of a color specific negativity in the VEP [16], [17].

By comparison, some fMRI analyses of color processing in human present evidence of the overall sensitivity of early visual cortex to chromatic processing [18], [19], [20]. Engel et al [18] using red/green patterned stimuli of varying saturation levels reported that color signals for perception are encoded in a large proportion of area V1/V2 neurons. Other fMRI experiments using hue discrimination protocols (based on the Farnsworth-Munsell 100 hue test) identify ventral occipitotemporal color sensitive areas. The most intensely activated areas were in the left and right collateral sulcus/fusiform gyrus and left mid-fusiform gyrus [21].

Optical imaging experiments in primate, capable of microscopic resolution, indicate chromatic concentrations in the blobs of V1 and in the thin stripes of V2 [22], [23], [24], [25], [26], [27], with systematic mapping of hue across the thin stripe [28] and fine scale mapping of hue in V1 [29]. While separation of chromatic and achromatic mechanisms have been demonstrated in both V1 and V2, it is not yet clear what is the exact relationship between surface or form color perception and the activation of early cortical regions.

Our previous work has demonstrated that the achromatic flash VEP possesses temporal non-linearities that naturally separate M-like and P-like contributions on the basis of interaction (or recovery) time between successive stimulations [30]. Also, using mixed chromatic and achromatic stimuli, we demonstrated a dissociation of chromatic (red) and achromatic contributions to the unstructured (surface) VEP [31]. We also showed that increasing contour length in a stimulus with equal areal content for black and white [32] resulted in an almost linear increase in first order kernel amplitude with contour length while the second order kernel did not increase with contour length. Hence we hypothesized that a similar separation of luminance and chromatic processing would exist with blue surface (diffuse) color stimulation.

**Figure 1:**
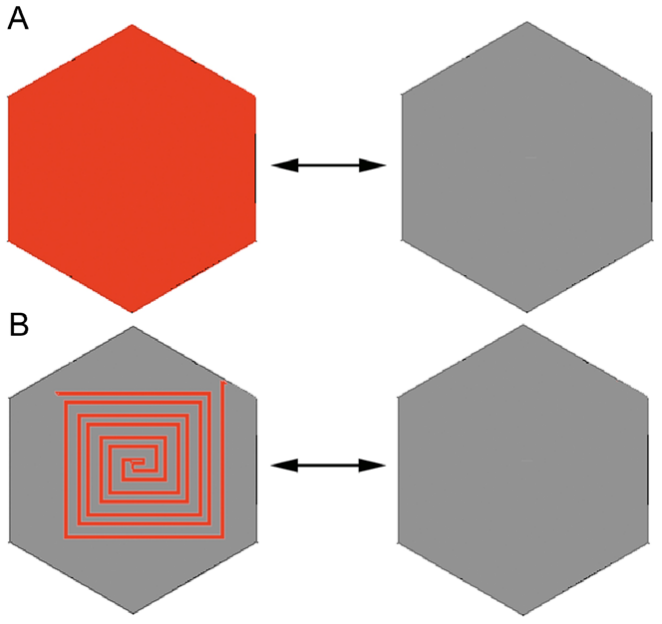
Binary exchange stimuli for the surface and form color responses. **A** Foveal hexagon of the multifocal stimulus exchanged between color (either red or blue) and grey. **B** Chromatic form stimuli consisted of a square spiral filled with the color of interest presented in appearance/disappearance mode against a background field of grey, exchanged with a featureless grey stimulus (same grey as for the form stimulus).

The description of a 4° unstructured central hexagon (see Figure 1) of the multi-focal stimulus, as a “surface color stimulus” might be queried in terms of contributions to the VEP from oriented receptive fields lying along its borders. Consideration of the whole sequence of multi-focal stimuli for one particular border between the central hexagon and any of it neighbours convinces otherwise [32]. During the stimulation sequence there are equal numbers of transitions from ‘no border’ (both of the hexagons are of the same color) to ‘border’ (central hexagon changes color) conditions as well as from ‘border’ to ‘no border’ conditions. Consideration of the nature of the kernel structure then suggests a direct cancellation of any edge contributions through subtraction, both for first and second order kernels.

The primary purpose of the present study was to compare the nonlinear temporal structures of color VEP responses when the stimuli contained form (pattern) and when they contained no form (diffuse or surface color), and to determine whether both forms of processing demonstrate a separation of luminance contrast and chromatic processing. On the basis of the early VEP recordings [12] we expected only weak chromatic response for the form stimulation condition and we expected that the blue diffuse responses would mimic those with red stimulation and demonstrate a luminance/chromatic separation. Six experiments were performed to investigate the chromatic surface and form VEPs under conditions of desaturation of red and of blue stimulation at constant luminance contrast, and also under isoluminant conditions (color/grey stimulation) for a range of hues from red to blue.

## Results

In terms of protocol, the surface and form stimulus presentations are similar - both involve the appearance of a red or blue image from a grey comparison hexagon - in one case, an unstructured or diffuse colored hexagon, in the other case a grey hexagon containing a red (or blue) contour (Figure 1). In addition, the ability to measure temporal recovery of these neural systems is provided through pseudorandom binary stimulus sequences.

**Figure 2:**
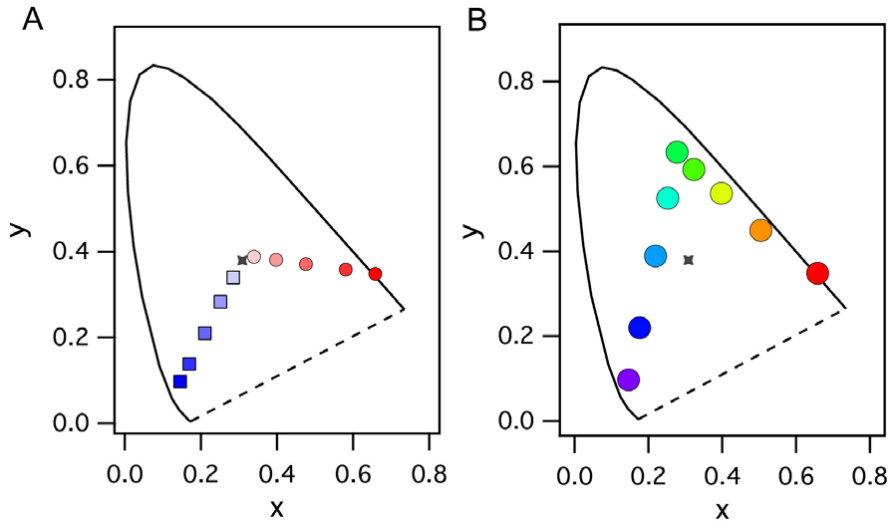
Chromaticity information (CIE1931) for the desaturation series and the hue dependence series. **A** Both the Red and Blue desaturations show monotonicity from the primary saturated colors on the outside of the curve towards the Grey point (cross). The luminance of color was maintained at 35 cd/m^2^ while the grey stimulus luminance was 30% higher (46 cd/m^2^). **B** CIE 1931 xy coordinates for the hue dependence experiments (5 & 6).

**Figure 3:**
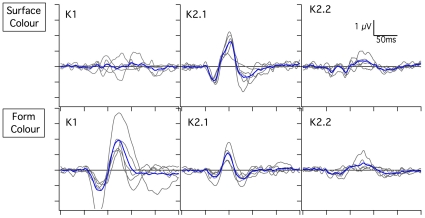
Comparison of non-linear VEP structure for Surface and Form color stimulation. The figure compares K1, K2.1 and K2.2 kernels for unstructured (surface color) stimulation and for form stimulation, presenting individual data from all participants as well as the mean waveform (blue line). The data shown come from blue stimulation at maximum saturation (with 30% luminance contrast). Clearly, most energy for surface color stimulation resides in the second order kernel slices, while most power for form stimulation resides in the first order kernel. The amplitudes of 2^nd^ order second slice (K2.2) responses are small.

The ranges of colors used in the desaturation surface color series (Expts 1 & 2), the desaturation form color series (Expts 3 & 4) and the isoluminant hue response series (Expts 5 & 6) are shown in Figure 2. Overall, the non-linear temporal structure of the physiological responses recorded from surface and form chromatic stimuli are very different, as demonstrated by the first and second order kernels (explained [33], and in the Methods section) (Figure 3). With diffuse (surface) stimulation, the first order responses are close to zero, with most of the VEP power residing in the second order responses, particularly K2.1. In contrast, for form stimulation there is a clear domination by the first order kernel. Thus, form and surface color responses have a different temporal structure.

### Surface Color

**Figure 4:**
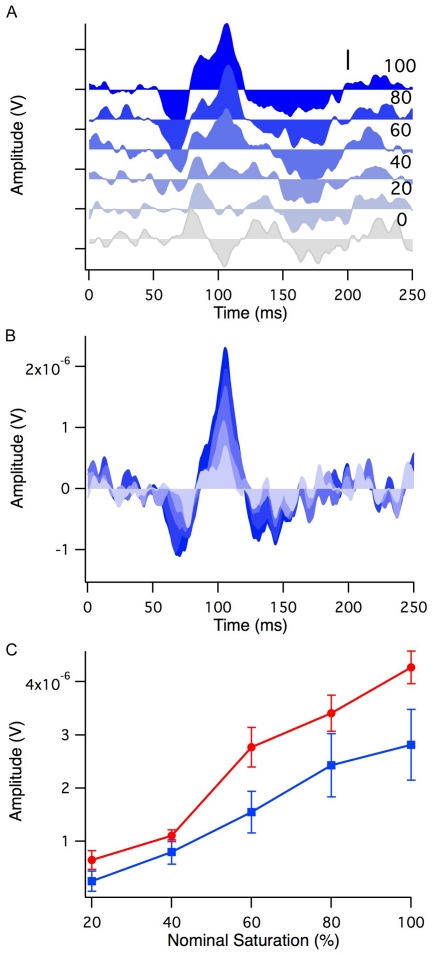
Evidence for the separability of a blue saturation from luminance contrast signals in the K2.1 surface color VEP. **A** Individual responses (participant LH) with constant luminance contrast and decreasing saturation from top to bottom (as indicated by the percentages to the right of the figure). **B** Results of subtraction of the 0% saturation (30% luminance contrast) response from the other waves of a. A unitary waveform emerges with almost linear dependence on saturation (LH). Scale bars  = 500 nV. **C** Mean P105 amplitudes, following subtraction, from the 5 participants as a function of saturation (% maximum). Also shown is the red surface color desaturation curve for the corresponding peak.

As expected from our previous investigations of surface color stimulation with red versus grey colors [31], the blue saturation dependent signal separates from that generated by luminance contrast. This can be clearly seen in Figure 4, which shows the waveforms from a single participant (LH) at constant luminance contrast, but variable color saturation for blue. The achromatic response (bottom trace Figure 4a) shows a distinct positive (P80)/negative (N105) waveform that remains partly visible until swamped at high saturation of blue color. A blue saturation dependent signal was revealed by subtracting the achromatic response from all of the other waves. A unitary waveform with almost linear dependence on saturation emerges for the P105 amplitudes of the K2.1 kernels (Figure 4b). A red-dependent component was also extracted from the red desaturation series at constant luminance contrast, as described in Klistorner et al (1998) [31]. Following subtraction of the achromatic waveform from the other waveforms, a saturation dependent signal was obvious.

This finding was replicated across the 5 participants with linear regression on the P105 peak amplitude (measured zero to peak) showing a strong correlation (Blue: adjusted R^2^  = 0.675, p<.0005; Red: R^2^  = 0.718, p<.0005; see Figure 4c). In both first and second order kernels, the major peak of the red (or blue)-sensitive (color) component had a longer latency than that for the achromatic response with a lag of 20–30 ms (consistent with previous research [14], [34], [35]), and a triphasic negative-positive-negative waveform.

### Form Color

**Figure 5:**
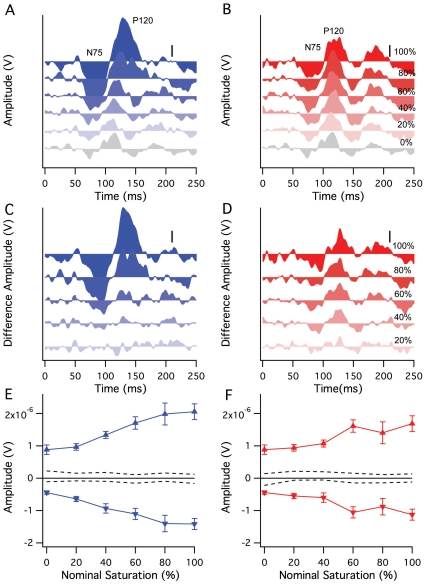
Effects of desaturation on form color responses. **A, B** Desaturation series (100%,80%, 60%,40%, 20%, 0%) at constant luminance contrast for Blue and Red Form Color stimulation (Participant MH – first order kernel, K1). It is clear that the while there is some saturation dependence, the waveforms for the maximally saturated and zero saturated (achromatic) form stimulation have similarities in profile, particularly for the N75 and P120 peaks. In this participant, a distinct positivity is observable in the red desaturation series at around 180 ms, but not in the blue desaturation series. Scale bar  = 500 nV. **C, D** Subtraction of the achromatic waveform from the desaturation series. **E, F** Means (±1 SE) across participants of the amplitudes of the N75 and P120 peaks of the difference waveforms for the red and blue desaturation series, with dotted lines showing noise estimates.

As distinct from the obvious effects of surface color desaturation on the second order waveforms generated by an individual participant, color desaturation for the appearance of the square spiral form stimulus affects the waveforms to a lesser degree, for both red and blue desaturation sequences. This is illustrated for participant MH in Figure 5.

While all K1 waveforms show some dependence both in latency and amplitude on the amount of saturation of color (with constant luminance contrast), N75 and P120 waveforms are immediately recognizable for all waves recorded, from achromatic to maximally saturated. The effects of subtracting the achromatic stimulation condition from the rest, as was done for surface color (Figure 5C, D), results in a confusion of small peaks at low saturation as distinct from specific amplitude dependence at low saturation seen in Figure 4. The positivity at around 180 ms for red desaturation in Figure 5B but not for blue desaturation (Figure 5A), is not consistently observed across participants.

Curiously, for maximum saturation in the blue series, there appears to be relatively strong activation, more so than for saturated red color. However, the mean difference graphs for both the N75 and P120 peaks of the form color response only show approximately 1 µV difference for 100% saturation compared with 3–4 µV for the maximal surface color difference response.

### Different chromatic dependence of surface and form color VEP, at isoluminance

**Figure 6:**
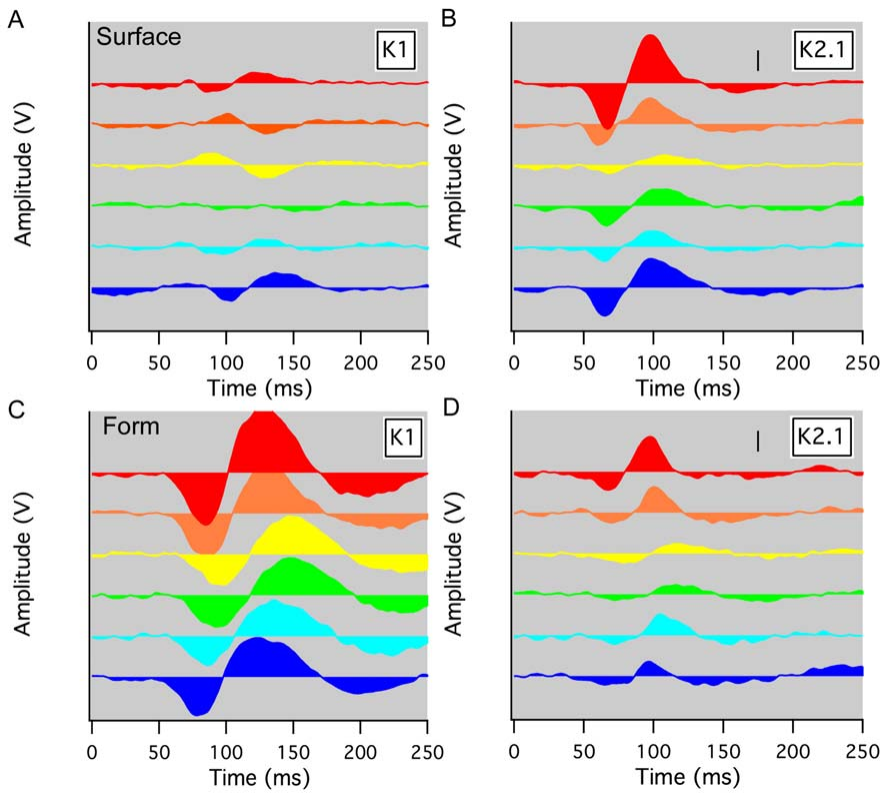
Spectral responses of form and surface color responses at isoluminance. First (K1) and second order (K2.1) responses to surface color stimulation (**A, B**) and form color stimulation (**C, D**) at isoluminance for 6 different hue values against grey color (Participant TY, scale bar 500 nV). There is a clear difference in the hue dependence of the form versus surface color response, especially for mid-spectral regions (eg Yellow-Grey stimulation) – surface color response almost vanishes while form color response is very robust.

In Experiments 5 and 6, the surface and form color responses for isoluminant stimulation (color/grey) were compared using 9 hue values (red, orange, yellow, lime, green, green cyan, cyan, sky blue, blue). Systematic differences in the spectral response can be seen between the main waves for surface (K2.1) and form appearance (K1) VEP (see Figure 6). Again the major power in the VEP resided in the first order response for form color and in K2.1 for the surface color response.

Several differences are obvious from Figure 6. First is the manifest difference in weighting of response within first and second order kernels. The second difference is the fact that though many fewer colored pixels are contained in the form compared with the surface stimulus, the response is larger. The surface color K1 response is quite variable and small. For the K2.1 response across stimulus hues (Figure 6B), there are very clear minima for the N65 and P100 peaks. These peaks nearly vanish for Yellow-Grey isoluminant stimulation.

The form color response across hues is characterized by prominent responses for all colors - the prominent form color N80-100 peak obviously does not vanish at Yellow, neither does the P115-140 (see Figure 6C). There is a lesser second order K2.1 response which resembles the surface color K2.1 response, though weaker. Secondly, the implicit time for the Form color response P115-P140 (see Figure 6C) increases markedly around Yellow-Grey stimulation and then returns for Blue-Grey stimulation to about the same level as for Red-Grey stimulation. The latency fluctuates by nearly 25 ms across the different hues used for stimulation, with relatively little change in amplitude.

**Figure 7:**
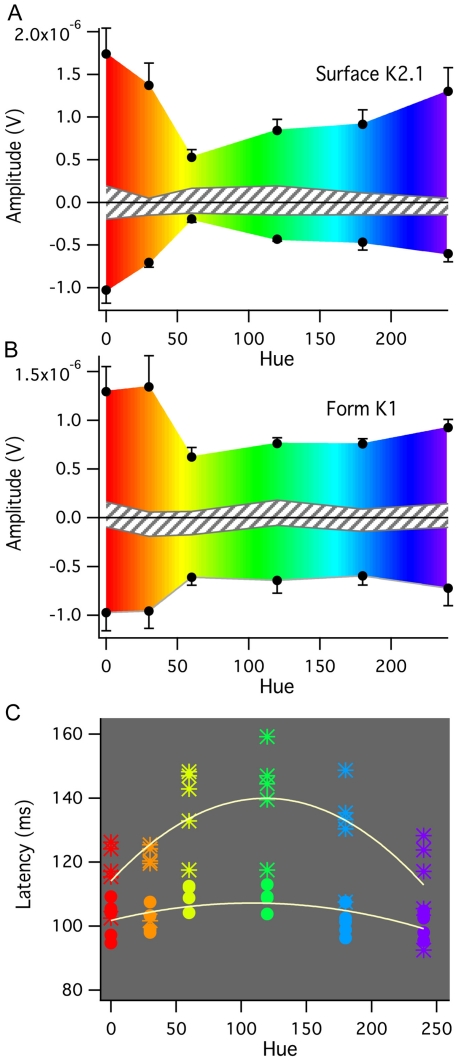
Comparison of Form (K1) and Surface (K2.1) color VEPs across Hue values from Red to Blue. Mean amplitudes and latencies of the major peaks for form and surface color stimulation plotted as a function of nominal Hue. The different colored stimuli are all isoluminant with the Grey stimulus used in the binary exchange. **A** N65 and P100 amplitudes for Surface color K2.1 plotted against Hue value. **B** N80-100 and P115-140 Form color K1 kernels plotted against Hue value. Data is presented as means and standard errors, with an estimate of the noise in the recordings indicated as the striped bar passing across the middle of the graphs (estimated from the maxima and minima found in first 30 ms of recording). **C** The individual latencies of the P100 wave for Surface K2.1 (dots) and P115-140 Form K1 (stars) color stimulation plotted against stimulus nominal Hue. A parabolic fit function with respect to hue demonstrates the clear latency increase around yellow for Form K1 compared with Surface K2.1 major responses.

The major surface color (K2.1) and form color (K1) responses were subject to further analysis. The N65 and P105 surface (K2.1) latencies and amplitudes and the N80-100 and P115-140 latencies and amplitudes were extracted and means and standard errors calculated. The resulting graphs for the Surface K2.1 kernels and the Form K1 kernels are shown in Figure 7. The N1 amplitude for Yellow-Grey surface color stimulation (K2.1 response) is not different from the noise estimate based on maxima and minima recorded for all participants in the epoch 0–30 ms – a time earlier than cortical VEP activations (Figure 7A). The P1 amplitudes of this response also dip strongly for Yellow hue – to less than 30% of end-spectral amplitudes. By comparison, for form color stimulation, the first order (K1) N1 and P1 amplitudes are roughly constant and finite for Yellow through to Blue (Figure 7B). In addition, the latency behaviour of the P1 peaks of the surface K2.1 and form K1 responses were plotted across the hue parameter (commonly found in color software (with range 0–360) (see Figure 7C, indicating the hues and parabolic fits to the data). The Form color K1 response shows a strong retardation of latency around Yellow – Green of almost 25 ms compared with end spectral values (as noted above for Figure 6). The surface color K2.1 latencies showed little retardation (∼5 ms) around Yellow-Green.

## Discussion

This investigation has shown that the temporal structure of form appearance chromatic VEP is distinct from that for the surface chromatic VEP. The first order kernel response for surface color stimulation is close to zero, while the main contribution for its temporal structure is to the first slice of the second order response, whereas the major contribution of form color stimulation is to the first order response, with a lesser second order first slice response. In itself, this result suggests the different weighting of contribution of the various neural receptive field types to the form and surface color VEPs. Perhaps this is not totally unexpected – color fusion frequencies, indicative of an adaptive non-linearity are quite slow (10–15 Hz), while recognition and discrimination of complex (colored) stimuli presented in a serial fashion is very rapid [36], with foreground/background segmentation of form requiring less than 10 ms asynchrony [37]. Also, oriented receptive fields with spatially segregated excitatory regions (eg double-opponent cells [11] of V1) are likely to respond to form stimuli (of the preferred orientation), but unlikely to produce strong response to diffuse (surface) stimulation covering the whole receptive field.

Secondly, the surface color dependent VEP is easily separable from the luminance contrast dependent component both for red color saturation and for blue color saturation stimulus series, with the saturation and achromatic waveforms manifestly different in appearance (Figure 4). Such a linear superposition between saturation and achromatic waveforms does not occur as simply for form appearance VEP for either red or blue color stimulation. The achromatic form VEP waveform resembles considerably that of chromatic form VEP (Figure 5), and while there is certainly variation in amplitude and latency with saturation, an analogous subtraction process (as shown in Figure 4) does not result in easily interpretable data. Thus one is tempted to interpret the form VEP responses as more weakly color dependent, on the basis that apparently the same neurons are responding to chromatic as well as achromatic stimulation. Such a claim would require more detailed testing, but does conform with the early primate reports of chromatic sensitivity of interblob orientation selective neurons in V1.

Thirdly, the spectral dependence of surface and form color is rather different, with the surface color N1 amplitude almost vanishing with yellow-grey isoluminant stimulation whereas form color VEP still has a robust recordable signal well above noise level. The latency of the main form color VEP P115-140 positivity shows a large delay around the yellow and green part of the spectrum, whereas the surface color P100 latency remains stable across wavelengths. While chromatic aberration of form stimuli can result in luminance contrast artifact [38], the greatest difference observed here is in the yellow region of the spectrum, where color/grey chromatic aberration effects would be least. Some variability across the spectrum would be expected, particularly as the cone contrast of the stimuli varies, despite all colors being isoluminant. However, the differences observed between form and surface color waveforms in the yellow region of the spectrum cannot be explained as identical stimulus parameters were used.

Previously, we established that a red color desaturation signal, additively dissociable from achromatic contrast responses was present in normal trichromatic vision in humans, but absent in dichromats [31]. We reported then that the major peak of the red color component had a longer latency than that for the achromatic response by 20–30 ms, consistent with previous research [14], [34], [35]. Very similar results have been found here. More importantly, there is a strong similarity between the red and blue sensitive components of the surface VEP (Figure 4) despite the fact that the underlying afferent neural circuitry for red and blue sensitive processing is very different [2]. The similarity could derive from the fact that both red and blue stimuli strongly activate the V1 blobs, which presumably contribute to the recorded VEPs. Dow and Vautin (1987) in single cell recording from primate V1 compared color properties of columns of single cells with non-oriented and oriented receptive fields, revealing that cortical middle layer cells in non-oriented type penetrations showed poor responses to white light, and color preferences for end-spectral wavelengths, i.e., red or blue. This segregation of color and orientation sensitivity is also beautifully demonstrated with optical imaging techniques [6], with the chromatic blobs aligning well with the cytochrome oxidase defined blobs.

The comparison of surface and form stimulation at isoluminance across the hue spectrum from red to blue demonstrates both similarities and differences. Both stimulus types demonstrate weaker responses in the yellow part of the spectrum: for surface color stimulation, this is a strong minimum, however for form color stimulation, while the response to red hues is greater, the response from yellow through to blue is almost constant in terms of mean amplitudes. This is to be expected as L and M cone contrasts are close to zero for Yellow/Grey stimulation, and also because end-spectral sensitive cells respond quite well to stimuli that are equiluminant, while midspectral cells fail to respond, or respond only very weakly to such stimuli, requiring higher luminance levels for optimal response [39]. The recent experiments of Johnson et al [11] may provide some explanation. They showed, in recording from primate area V1, that the majority of double-opponent cells are orientation selective to luminance contrast as well as to achromatic patterns. Also, the responses of such cells were shown to be contrast dependent, whether chromatic or achromatic. These double-opponent cells could thus provide the basis for the form dependent VEP that shows a qualitatively similar waveform structure to chromatic and achromatic stimulation. Interestingly, Friedman et al [40], in addition to reporting edge-sensitive color-selective cells (presumably double-opponent), reported a smaller population of V1 color surface cells that were not particularly sensitive to contour. These may form the basis of the color surface VEP response that we recorded.

While an explanation based on the receptive fields recorded in primate single cell studies appears to accommodate the data well, the alternative hypothesis – that there is a single class of cells that are exhibiting these different temporal properties on the basis of stimulus differences, should be considered. The characteristics of the K1 responses are sufficiently different both in terms of presence and also in comparing latency of K1 and K2.1, to suggest a separate population of neurons that contribute. In addition the different spectral response at isoluminance of Form K1 versus Form K2.1 (see Figure 6) adds to the likelihood of separate neural contributors.

Do form color VEPs for stimuli have a component due to the edges of the form plus a component (colored) from the interior of the form where the conditions are unchanging? Certainly the form stimulus condition (appearance) could be related to the surface as the appearance of color, constrained by the boundary. This is partially answered by an achromatic non-linear VEP study looking at the effect of increasing contour length in a stimulus with equal areal content for black and white [32]. Here it was noted that the first order kernel responses showed a significant increase in amplitude with total contour length. In comparison, the amplitudes of the first and second slices of the second order kernel did not increase with contour length. It was noted there that the edge dependent component had little second order power, and that the constant, non-zero responses of the higher order kernel components may have reflected the contributions from the diffuse chromatic VEP, the lack of variation due to the constant total area of stimulation (of white and black). In relation to the current study, the second order K2.1 response recorded may be provided by the same neural system that generates the surface color response. It shows the same qualitative features as the surface color responses recorded, though rather smaller in magnitude (perhaps due to the smaller numbers of pixels being stimulated).

Given that two independent (mathematically, in terms of the Wiener kernel decomposition) color responses exist, we ask whether it is the surface color that provides the naming of the colors in stimuli while the colored form response is destined mainly for form processing? Moreover, do the two color processing systems interact and what are their contributions to responses under different recording situations? As an illustration, fMRI color publications typically use objects with both contour and color (gratings, patterns, etc) [18], [20], [41], [42], with perhaps only the hue discrimination fMRI experiments [21] that relate solely to the surface color signals reported here. In addition there are classes of experiments where both surface, pattern and color of objects are manipulated [43], [44].

## Materials and Methods

Five color normal participants were used in the study. The protocol and informed consent procedure were approved by the Swinburne University Human Research Ethics Committee and informed, written consent was obtained from all participants. All subjects were given a routine visual examination in which it was assessed that clear vision of the stimulus screen would be obtained. The color vision of the clinically normal participants was tested using the Ishihara standard pseudo-isochromatic plate test.

Non-linear VEPs were recorded using the VERIS system for topographic and temporal analysis of evoked potentials (Electro-Diagnostic Imaging, San Mateo; [33]). The VERIS multifocal system is based on the Wiener kernel expansion and utilizes a deterministic pseudo-random binary exchange at each of a number of sites (19 sites used in this study) of the visual field. First and higher-order kernels were computed but only the first and the second-order kernels were analysed. These are well explained in the paper by Sutter [33]. The first-order response can be thought of as the summed responses (R1) to stimulus one minus that (R2) to stimulus two, i.e. 0.5*(R1 − R2), while the first slice of the second-order response represents the comparison for consecutive display monitor frames containing a transition to those where no transition occurred, i.e. 0.25*(R11 + R22 − R12 − R21). Here, R11 represents the response to two consecutive frames of stimulus 1, R12 the response to stimulus 1 followed by stimulus 2, and so on. While the first slice of the second-order response (K2.1) relates consecutive frames, the second slice (K2.2) compares responses with an extra intervening frame (summed over all stimulus polarities). Thus the first slice represents the interaction present at a time scale of 15 ms, the second slice interaction at a time scale of 30 ms (one intervening frame) and so on. The wide spectrum of temporal frequencies used undoubtedly stimulates elements of both the faster luminance and slower color pathways.

The VEP was recorded using gold cup electrodes (Oz referenced to Fz with single ear ground). The signal was amplified 100,000 times and band-pass filtered between 3 and 1000 Hz. The data sampling rate was 1000 Hz. The m  = 14 stimulus sequence used (divided into eight slightly overlapping segments) was of total length 2^14^ − 1 frames, corresponding to a 4 min recording period. The distance to the screen was 50 cm. A standard stimulus of 19 equal-sized hexagons was used in all experiments (displayed on a 19 in. La Cie high luminance CRT monitor). The central hexagon subtended 4°, and was the only region used in subsequent analysis.

All hexagons were alternated in pseudo-random sequences between color and grey. While data from only the central hexagon were analysed, the peripheral hexagons formed an effective surround of the same mean luminance, chromaticity and temporal characteristics. This has the effect also of removing from the averaged VEPs contributions of the hexagonal contour forming the boundary of the central element. For example, with red/grey stimulation, if the central hexagon is red, the neighbouring patch to the left is either red or grey with a 50% chance. Similarly, when the central hexagon is grey, the neighbouring patch to the left is either red or grey with a 50% chance. Thus, in the simple subtraction for the K1 estimation, the contributions from edge dependent mechanisms will tend to cancel.

### Expts 1 & 2: Surface color desaturation at constant luminance contrast

Binary exchange between colored hexagons (see Figure 1a), either red (Expt 1) or blue (Expt 2) with luminance 35 cd/m^2^, occurred with a (brighter) grey hexagon (luminance 46 cd/m^2^). Luminance contrast was maintained as the nominal saturation was reduced from 100%, to 80%, 60%, 40%, 20% and 0% (achromatic) in successive acquisitions, where the percentages refer to the HSV saturation coordinate.

The luminance characteristics of the screen were measured using a Tektronix J6523 1° narrow angle luminance probe, prior to each recording, while colorimetric information was provided using a Monaco OPTIX colorimeter (X-rite) and a Red Tide USB650 (Ocean Optics) spectrometer (see Figure 2). Spectrometer output was normalized to the measured luminance, resulting after integration across wavelengths in the CIE (1931) XYZ coordinates for each color and also in the La*b* color space coordinates (using Color Converter, version 2.3, http://www.rmimaging.com. In addition, cone contrasts (color vs grey) were calculated from the spectrometric data [45]. The measured luminance, and the saturation values, La*b* coordinates and cone contrasts of the Red and Blue desaturation series are displayed in Table 1.

**Table 1:** Desaturation Stimulus values: Hue, Saturation, Value parameters and measured luminance, saturation, CIE 1931 (x,y), and cone contrasts for the Blue and Red Desaturation series against the Light Grey Stimulus.

Color	HSVcoordinates	Lum (cd/m^2^)	Measured %Saturation(CIE 1931)	CIE(x,y)	Cone ContrastC_L_	Cone ContrastC_M_	Cone ContrastC_S_
Blue	(240,100,100)	35	87.8	(0.145, 0.097)	−0.172	0.309	8.120
	(240,80,78)	35	75.1	(0.169, 0.138)	−0.105	0.189	4.940
	(240,60,59)	35	52.9	(0.210, 0.210)	−0.049	0.089	2.314
	(240,40,45)	35	30.2	(0.252, 0.283)	−0.022	0.040	0.985
	(240,20,36)	35	12.6	(0.285, 0.339	−0.008	0.014	0.342
Red	(0,100,63)	35	100	(0.658, 0.348)	0.269	−0.484	−0.894
	(0,80, 57)	35	77.7	(0.581, 0.357)	0.200	−0.360	−0.671
	(0, 60,50)	35	47.7	(0.476, 0.370)	0.120	−0.215	−0.404
	(0, 40,41)	35	25.5	(0.398, 0.381)	0.061	−0.111	−0.219
	(0,20,35)	35	9.1	(0.340, 0.388)	0.024	−0.044	−0.087
Grey Hi	(0,0,36)	46	0	(0.309, 0.379)	−	−	−

### Expts 3 & 4: Form color VEP desaturation at constant luminance contrast

Expts 3 & 4 were analogous form color VEP desaturation series, using the appearance of the square spiral stimulus of Figure 1b, with either red or blue form stimuli (nominal saturation 100%, 80%, 60%, 40%, 20%, 0%) with the grey comparison stimulus again set at 46 cd/m^2^.

### Expts 5 & 6: Spectral dependence of Surface and Form Color VEP at isoluminance

In Expts 5 & 6 we attempted to assess whether behaviour of form and surface color VEPs differed across the available gamut. To this end we investigated the first and second order responses to binary exchange of color/grey exchange across the spectrum from red through yellow and green to blue with isoluminant stimuli at the maximum available saturation. The stimuli used were either the surface color stimuli (Figure 1a) or the form color stimuli (Figure 1b). The stimulus parameters are set out in Table 2.

**Table 2:** Isoluminance Stimulus values: Hue, Saturation, Value parameters and measured luminance, saturation, CIE 1931 (x,y), and cone contrasts for the maximally saturated monitor colors against the isoluminant Grey stimulus.

Color Name	HSVcoordinates	Lum (cd/m^2^)	CIE(x,y)	Cone ContrastC_L_	Cone ContrastC_M_	Cone ContrastC_S_
Blue	(240,100,100)	35	(0.146, 0.097)	−0.172	0.309	8.120
Sky Blue	(210,100,58)	35	(0.176, 0.219)	−0.096	0.173	2.304
Cyan	(180, 100, 35)	35	(0.219, 0.389)	−0.069	0.123	0.221
Green/Cyan	(150,100,36)	35	(0.252, 0.524)	−0.060	0.108	−0.476
Green	(120,100,37)	35	(0.278, 0.634)	−0.056	0.101	−0.823
Lime	(90,100,35)	35	(0.323, 0.593)	−0.031	0.055	−0.819
Yellow	(60,100,31)	35	(0.398, 0.537)	0.017	−0.031	−0.848
Orange	(30,100,47)	35	(0.505, 0.450)	0.109	−0.196	−0.876
Red	(0,100,63)	35	(0.658, 0.348)	0.269	−0.484	−0.894
Grey Lo	(0,0,29)	35	(0.310, 0.383)	−	−	−
